# Initial Study Using Electrocardiogram for Authentication and Identification

**DOI:** 10.3390/s22062202

**Published:** 2022-03-11

**Authors:** Teresa M. C. Pereira, Raquel C. Conceição, Raquel Sebastião

**Affiliations:** 1 Departamento de Física, Faculdade de Ciências, Universidade de Lisboa, Campo Grande, 1749-016 Lisboa, Portugal; teremcpereira@gmail.com; 2 Instituto de Biofísica e Engenharia Biomédica, Faculdade de Ciências, Universidade de Lisboa, Campo Grande, 1749-016 Lisboa, Portugal; 3 Departamento de Electrónica, Instituto de Engenharia Electrónica e Informática de Aveiro, Telecomunicações e Informática, Universidade de Aveiro, 3810-193 Aveiro, Portugal; raquel.sebastiao@ua.pt

**Keywords:** biometrics, electrocardiogram, feature extraction, classification algorithms, comparative analysis

## Abstract

Recently, several studies have demonstrated the potential of electrocardiogram (ECG) to be used as a physiological signature for biometric systems (BS). We investigated the potential of ECG as a biometric trait for the identification and authentication of individuals. We used data from a public database, CYBHi, containing two off-the-person records from 63 subjects, separated by 3 months. For the BS, two templates were generated: (1) cardiac cycles (CC) and (2) scalograms. The identification with CC was performed with LDA, kNN, DT, and SVM, whereas a convolutional neural network (CNN) and a distance-based algorithm were used for scalograms. The authentication was performed with a distance-based algorithm, with a leave-one-out cross validation, for impostors evaluation. The identification system yielded accuracies of 79.37% and 69.84% for CC with LDA and scalograms with CNN, respectively. The authentication yielded an accuracy of 90.48% and an impostor score of 13.06% for CC, and it had an accuracy of 98.42% and an impostor score of 14.34% for scalograms. The obtained results support the claim that ECG can be successfully used for personal recognition. To the best of our knowledge, our study is the first to thoroughly compare templates and methodologies to optimize the performance of an ECG-based biometric system.

## 1. Introduction

Nowadays, there is a variety of real-world applications that rely on recognition systems to protect and guard ourselves, our information, or our belongings. Several still depend on traditional systems based on extrinsic entities or knowledge, such as cards, keys, pins, or passwords. However, these traditional mechanisms present several usability and security problems. Hence, surrogate representations of identity no longer suffice.

As a result, there has been a shift of interest toward the field of biometric recognition, which refers to the automatic identification of people based on their distinctive physiological (e.g., face, fingerprint, iris, hand geometry) and behavioral (e.g., gait, signature, keystroke) characteristics [1]. The most common biometric trait is the fingerprint, and while this is a significant step forwards, there are still problems related to fingerprint usability and reliability, as the acquisition of a fingerprint is often of poor quality and can also be easily circumvented by a skilled specialist.

Since the electrocardiogram (ECG) is a signal originated internally and unique to each person, it has the potential to be a reliable source for biometrics [2,3,4,5]. Current challenges include extracting relevant and reliable features from ECG signals and designing accurate models for template matching, protecting an individual against identity attacks. In this paper, the potential of using ECG-based information as a biometric trait for identification and authentication of individuals was investigated.

### 1.1. Objectives and Contributions

We address some of the limitations of existing research regarding the use of ECG signals for biometric identification and authentication. The ECG data used in our paper were taken from the Check Your Biosignals Here initiative (CYBHi) database [6,7], which overcame some of the limitations of previously reported data collection processes. Firstly, Silva et al. [6] used an off-the-person approach to collect ECG signals at the fingers using dry electrodes, which is a less intrusive approach than on-the-person, which requires the placement of electrodes in the chest and/or arms and legs. The proposed acquisition configuration has many potential applications, since it can be easily integrated into real-world scenarios. Secondly, ECG in CYBHi was collected from the same participants over two sessions, which were separated in time by three months. This, besides allowing examining the usability and uniqueness, also allowed analysis of the stability of human ECG for biometric recognition tasks, as will be detailed in Section 1.2.

Another challenge with using ECG data for biometric authentication and identification is that there is still no consensus on which features and classification methods lead to better performance. Thus, the present work used two biometric templates based on different types of features and tested different approaches and methods to perform template matching in order to find the optimal combination for biometrics identification and authentication.

Hence, this research aims to achieve the following:

- Demonstrate that the nature of ECG is sufficiently personal to be used in recognition systems;
- Investigate if the ECG is sufficiently stable over time for the system to perform recognition of individuals multiple times;
- Show that off-the-person acquisitions have the potential to be used in biometric systems;
- Perform a comparative analysis between different features and template matching methods to find optimal solutions.

### 1.2. Biometrics

Biometrics is defined by the International Organization for Standardization as the “automated recognition of individuals based on their behavioral and biological characteristics” [8]. They are present in our daily lives either for personal identification or authentication. In biometric authentication, the system validates the claimed identity of a particular person, whereas in biometric identification, the system finds out who the person is without any previously claimed identity.

Distinctive characteristics have been used as biometric traits, such as fingerprint, face, iris, hand geometry, voice, signature, and gait. ECG has recently been proposed as a biometric trait due to its hidden nature and inherent liveness information. Moreover, the ECG is believed to be unique and different from one person to another, having the potential to distinguish different individuals. Most existing literature focused on proving the feasibility of an ECG as a biometric, showing the different characteristics that a biometric trait must have [9]:

- Uniqueness: The nature of ECG is sufficiently personal to be used in high-performance identity recognition systems.
- Stability: Demonstration of stability requires data to be collected from the same individual over a sufficiently long period of time.
- Collectability: Nowadays, several minimally invasive and portable devices can record ECG using electrodes placed only on the chest. Moreover, there are off-the-person approaches for signal acquisition at the wrists, hands, or fingers.
- Performance: The performance of a biometric system depends on several aspects, such as the signal acquisition process, the quality of the signal, the pre-processing procedures, the selected features, the template used, and the matching algorithm [9].
- Acceptability: With the introduction of reliable minimally invasive ECG devices, there have been more opportunities to create ECG-based biometric systems that are less invasive and, consequently, more socially accepted.
- Circumvention: All biometric systems are subject to attacks, which try to corrupt the system with an artifact or contraption. However, forging an ECG recording is a much more complex procedure when compared to forging other biometric traits.

### 1.3. Biometric Systems

A biometric system (BS) is a system that performs a biometric task based on three main stages: data acquisition, data processing, and pattern matching.

Regarding data acquisition, a BS requires two types of templates: (1) enrollment template and (2) presentation template. The enrollment template is generated when a user registers for the first time, and it is stored in the database. The presentation template is generated every time a user tries to gain access [10].

Once the biometric signals are captured, they are transformed, using signal processing techniques into reference templates that will be used to distinguish the individual. This may involve several steps, such as artifacts detection, signal filtering, signal segmentation, amplitude and time normalization, outlier detection, and feature extraction. In this context, the extracted features can be fiducial, non-fiducial, or partially fiducial, depending on their nature. Concerning a biometric recognition algorithm, a biometric algorithm takes the features from the stored enrollment template, along with the features extracted from the presentation template, and compares them to generate a score indicating the likelihood that both are from the same person. The algorithm can support one or two crucial functions: authentication and/or identification. Authentication involves confirming or denying a person’s claimed identity. The system performs a one-to-one comparison of the acquired biometric data with the stored information associated with the claimed identity. In identification, the biometric system must establish a person’s identity by performing a one-to-many comparison of the acquired biometric data with the information of all the individuals in the database. The identification mode does not require the user to claim an identity.

### 1.4. Electrical Activity of the Heart

**Figure 1:**
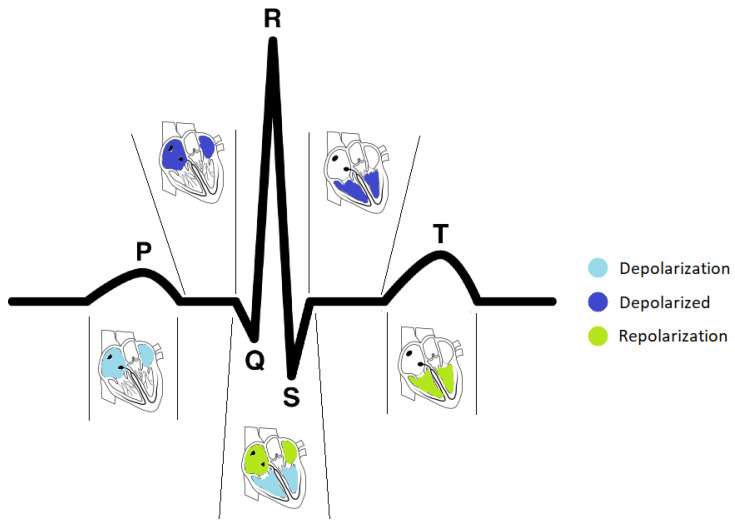
The sequence of depolarization and repolarization events in the heart and their relationship with the different heartbeat waveforms in an ECG signal (adapted from [12], figure kindly provided by João Ribeiro Pinto and Jaime Cardoso).

An electrocardiogram is a recording of the heart’s electrical activity. Each cardiac cycle comprises two phases, depolarization and repolarization, which are referred to in mechanical terms as contraction and relaxation. A typical ECG wave of a normal heartbeat, such as the one presented in Figure 1, consists of a P wave, a QRS complex, and a T wave.

The P wave is generated when the right and left atria are depolarized. Its amplitude usually is less than 300 µV, and its duration is less than 120 ms. The spectral characteristic of a normal P wave is usually considered to be low-frequency, below 10–15 Hz.

The QRS complex reflects the depolarization of the right and left ventricles. The first negative deflection of the QRS complex is the Q wave, the first positive deflection is the R wave, while the subsequent negative deflection is the S wave. Its duration may extend up to 250 ms, and its frequency content is considerably higher than that of the other ECG waves and is mainly focused in the interval 10–50 Hz. Since the QRS complex has the largest amplitude of the entire ECG waveform (sometimes reaching 2–3 mV), this complex is the first to be identified in any computer-based analysis [11].

Finally, the T wave occurs during ventricular repolarization and extends about 300 ms after the QRS complex. Atrial repolarization cannot usually be discerned from the ECG, since it coincides with the much larger QRS complex.

### 1.5. Paper Organization

This work is organized as follows. In Section 1.2 and Section 1.3, a brief overview of biometrics and the functioning of a biometric system is presented. Section 1.4 presents a description of an ECG signal. Section 2 consists of a review of the most relevant work in ECG biometrics. In Section 3, we present a description of the data and the proposed biometric authentication and identification systems based on ECG signals. Section 4 shows the results obtained for the identification and authentication systems. Section 5 presents the discussion of the results and Section 6 shows a summary and future work directions.

## 2. Literature Review

Previous studies on ECG-based biometric systems can be differentiated according to the design choices made with respect to data acquisition, feature selection, and template matching techniques. These are detailed in the following text.

### 2.1. Data Acquisition

The configurations used for ECG acquisition in biometrics research have significantly evolved over the years, mainly intending to overcome the major disadvantage of ECG as a biometric trait: acquisition acceptability.

In early ECG biometric research, on-the-person recordings from standard 12-lead or Frank leads were commonly used for the development of biometric algorithms [13,14,15]. Over time, some researchers started investigating the selective use of certain leads of these configurations, especially Lead I [16,17,18], because of its higher acceptability due to the possibility of placing the electrodes at the wrists, but also Lead II [19,20,21], or two chest leads [22,23]. Some researchers, such as Labati et al. [24] and Zhou et al. [25], opted for acquisitions without movement restrictions, with fewer electrodes, and with longer duration, using Holter systems to acquire ECG signals during several hours while the subjects are performing their daily basis activities. In off-the-person acquisitions, dry metallic electrodes replaced wet electrodes, their number was reduced to two or three, and their placement was commonly confined to the upper limbs, especially wrists, hands, or fingers [6,16,26,27]. Recently, some researchers have been improving off-the-person configurations by developing wearable technologies for ECG acquisition or embedding the sensors into ordinary objects. Some examples of this type of configuration are a computer keyboard equipped with a sensor for ECG acquisition [28], the Nymi band [29], which is a wearable wristband acquiring ECG using two metallic electrodes, and the CardioWheel, which is a steering wheel cover made of conductive leather [30].

Even though ECG signals obtained during normal resting conditions have been investigated in most studies, some researchers test the feasibility of ECG biometrics under different conditions, such as during changes in emotional and mental states [31], during physical exercise [32], and in individuals with cardiovascular disorders [33]. All of them proved that biometric recognition is feasible under such conditions, even though the performance is worse when compared to the performances obtained with ECG data acquired in normal resting conditions.

### 2.2. Feature Selection

Regarding feature selection, existing approaches can be broadly classified as fiducial, partially fiducial, and non-fiducial [9]. Algorithms based on fiducial features extract points of interest within the heartbeat wave, which are then used to extract latency and amplitude features. Most of the research in the field of ECG-based biometrics uses fiducial-based features [18,19,21,31,32,34]. Algorithms based on non-fiducial features extract discriminative information within the ECG waveform, which may not have a direct physiological relationship with the reference points in the heartbeat waves. Some non-fiducial methodologies proposed in the literature use Wavelet Transform [26], Discrete Cosine Transform, and Autocorrelation Coefficients [14,35]. Partially fiducial approaches rely on fiducial information only for ECG segmentation, using non-fiducial methods to create the feature vectors that form the biometric templates [17,36,37].

### 2.3. Template Matching

The template matching aims to accurately attribute one of the enrolled identities to the user, in the case of identification tasks, or accept or reject an identity claim, in authentication tasks. In the case of identification, the template matching usually consists of a classification process in which the classifiers are trained with the stored templates and tested with the presentation templates.

The most commonly used classifiers are SVM [23,35,38], k-nearest neighbors classifiers (kNN) [3,15,39], or neural networks [13,27,37]. Previous literature support that SVMs, kNN, and neural networks are, in fact, the most valuable methods for biomedical applications, such as ECG analysis [40] and the classification of ECG and EEG features for the detection of various disorders [41,42].

For authentication, the acceptance or rejection of the identity claimed is generally based on a distance-based matching, which was compared within a reference threshold. Within the several distances used, the Euclidean distance was, by far, the most popular [14,20,38,43,44]. However, the following are also reported in the literature, such as the cosine distance [38], the Mahalanobis distance [19], the Wavelet distance [37], and the Gaussian log-likelihood [45].

### 2.4. Related Works

Biel et al. [34] used features directly outputted by an ECG medical acquisition device and performed decisions using Principal Component Analysis, obtaining an identification rate (IDR) of 100% with 20 subjects. Kyoso et al. [19] extracted 34 fiducial features and achieved 99.5% and 94.2% IDR, with three and nine subjects, respectively, using Linear Discriminant Analysis (LDA) for dimensionality reduction and Mahalanobis distance-based kNN for classification. Palaniappan et al. [17], Chan et al. [26], and Singh et al. [43] have used time-domain features, and they achieved an accuracy of 97.6%, 90.8%, and 99% for identification, respectively. Several non-fiducial approaches have been attempted, such as Fourier transform [32], discrete wavelet transform (DWT) [46], and autocorrelation coefficients (AC) [14], with a recognition rate from 77% to 100% for as many as 35 subjects.

Chan et al. [26] were the first researchers to explore the off-the-person approach for biometrics, with metallic electrodes on the thumbs, obtaining 89% of IDR. Coutinho et al. [47] acquired signals from hand palms, using a conductive mat next to a computer keyboard, and reached 99.5% IDR. Matta et al. [44] pioneered the continuous identification of 10 subjects, assessing identity every five seconds with 75% IDR, using AC and LDA for feature extraction and Euclidean distance-based kNN for classification.

Pinto et al. [48] investigated the influence of the quantity of training data on system performance. This approach was tested for identification and authentication tasks in two settings: firstly, using 70% of each subject’s data for training and 30% for testing; and secondly, using solely the first 30 s of data from each subject for training. The results were worse with the second setting for both tasks (IDR decreased from 94.6% to 70.9% and authentication Equal Error Rate (EER) increased from 2.66% to 11.8%).

Other aspects that have been explored in ECG biometrics address the effects of heart rate variability, using different leads, and long-term acquisitions. Fang et al. [22] and Zhang et al. [46] observed that using one lead renders significantly worse results than three leads; and using limb leads, such as I or II, decreases the performance compared to the use of chest leads V1 or V2, respectively. Ye et al. [23] observed that the performance, using DWT and Independent Component Analysis (ICA) features with SVM (with RBF kernel), applied to long-term signals, is consistently worse than those applied to short-term signals. Pathoumvanh et al. [20] verified that the IDR of their system, based on Convolutional Wavelet Transform (CWT) features and Euclidean distance-based kNN, decreased from 97% to 80% when using signals acquired after exercise. Moreover, Hwang et at. [49] proposed an authentication model based on 29 fiducial features and testing several machine learning classifiers. The performance of the models was evaluated with ECG on-the-person recordings of 15 subjects, which were collected under different physiological conditions, and the best model achieved an accuracy of 99.05% in the resting state and 88.14% in non-resting states. While the results are very promising, we would like to note that a relatively small database was used in that paper, along with on-the-person ECG acquisitions, which is limiting for mimicking realistic recognition systems.

## 3. Materials and Methods

**Figure 2:**
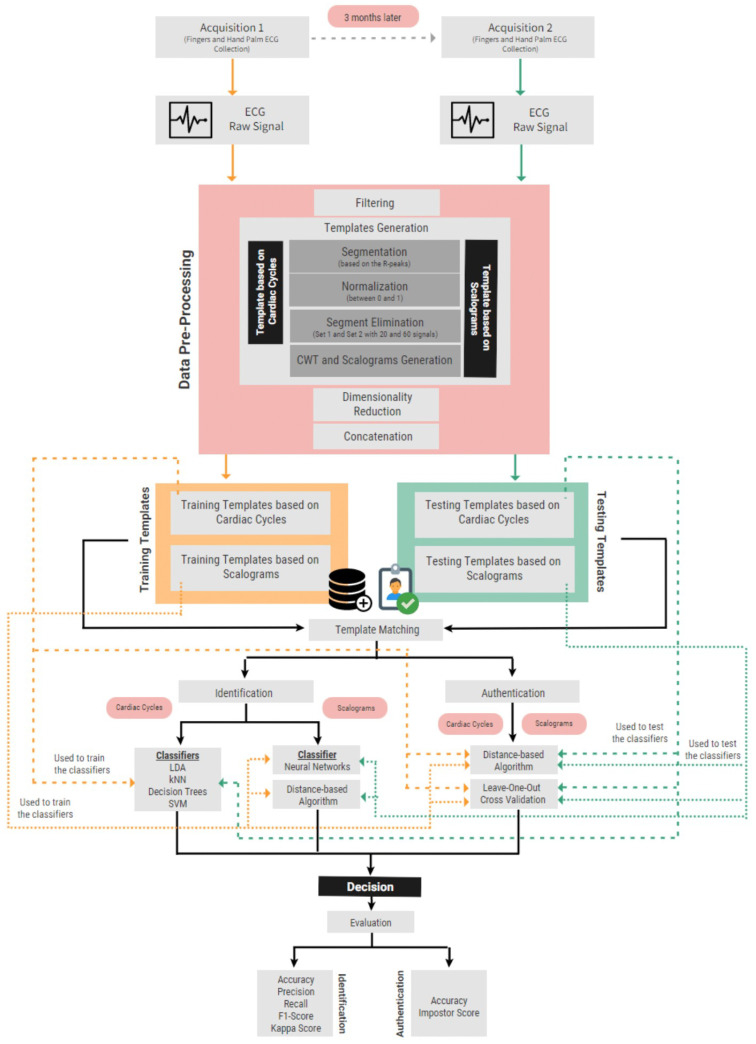
Flow chart of the proposed systems.

This section describes the design of the proposed biometric identification and authentication systems based on ECG signals. The suggested systems use a database with ECG signals collected with an off-the-person approach. Two different templates were used: cardiac cycles and scalograms. Finally, several classifiers and a distance-based approach were tested for template matching. Figure 2 illustrates the overall system design. The experiments were performed in Python (using the NeuroKit2 package [50]) and Matlab (MATLAB R2020b and Simulink R2020b).

### 3.1. Database Description

We used ECG data from a publicly available database, the CYBHi database, which followed an off-the-person approach for ECG acquisition at the fingers, with dry electrodes [6]. Raw biosignals were acquired with a biosignalsplux Researcher kit [51], with a bluetooth wireless biosignal acquisition unit. This device was used in a 12-bit resolution with 1 kHz sampling frequency configuration. A total of 63 subjects (nursing and health technologies students) were enrolled in the experiment and participated in two different acquisition sessions. The demographics showed 12 males and 49 females, with an average age of 20.68 ± 2.83 years old. None of the participants reported any health problems. The two data acquisition moments were separated by 3 months, and in both, the ECG was recorded in a sitting position for 2 min, at two fingers—one from the left and another from the right hand—with dry Ag/AgCl electrodes.

### 3.2. Data Pre-Processing

The pre-processing task performed in our paper, for both identification and authentication algorithms, consists of three steps: signal filtering, templates generation, and dimensionality reduction.

#### 3.2.1. Signal Filtering

In general, ECG signals present a noise component caused by respiration, electrodes impedance, and also powerline interference. In addition to these, since the signals were collected at the fingers, they may contain electromyographic interferences. In our paper, a 4th-order Butterworth bandpass IIR filter with cut-off frequencies of 0.5 Hz and 30 Hz was applied to the raw ECG in order to remove undesired frequencies and smooth the signal.

#### 3.2.2. Template Generation

After filtering the ECG, two different types of templates were generated: (1) template based on cardiac cycles, and (2) template based on scalograms of the cardiac cycles.

**Figure 3:**
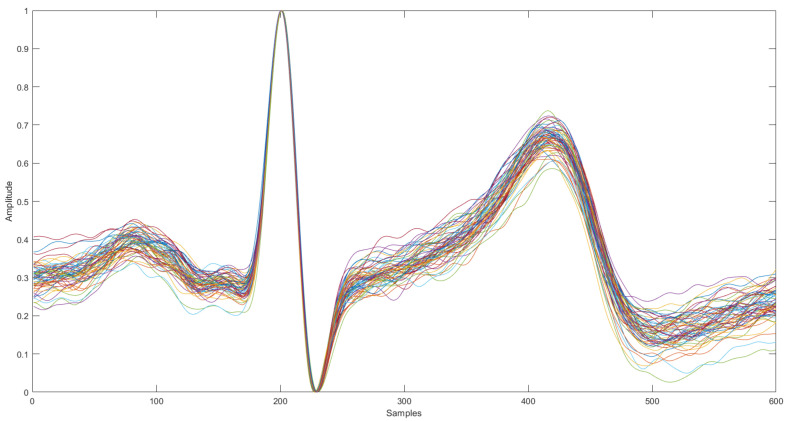
ECG variation within a single subject after performing segment elimination (60 waveforms).

**Figure 4:**
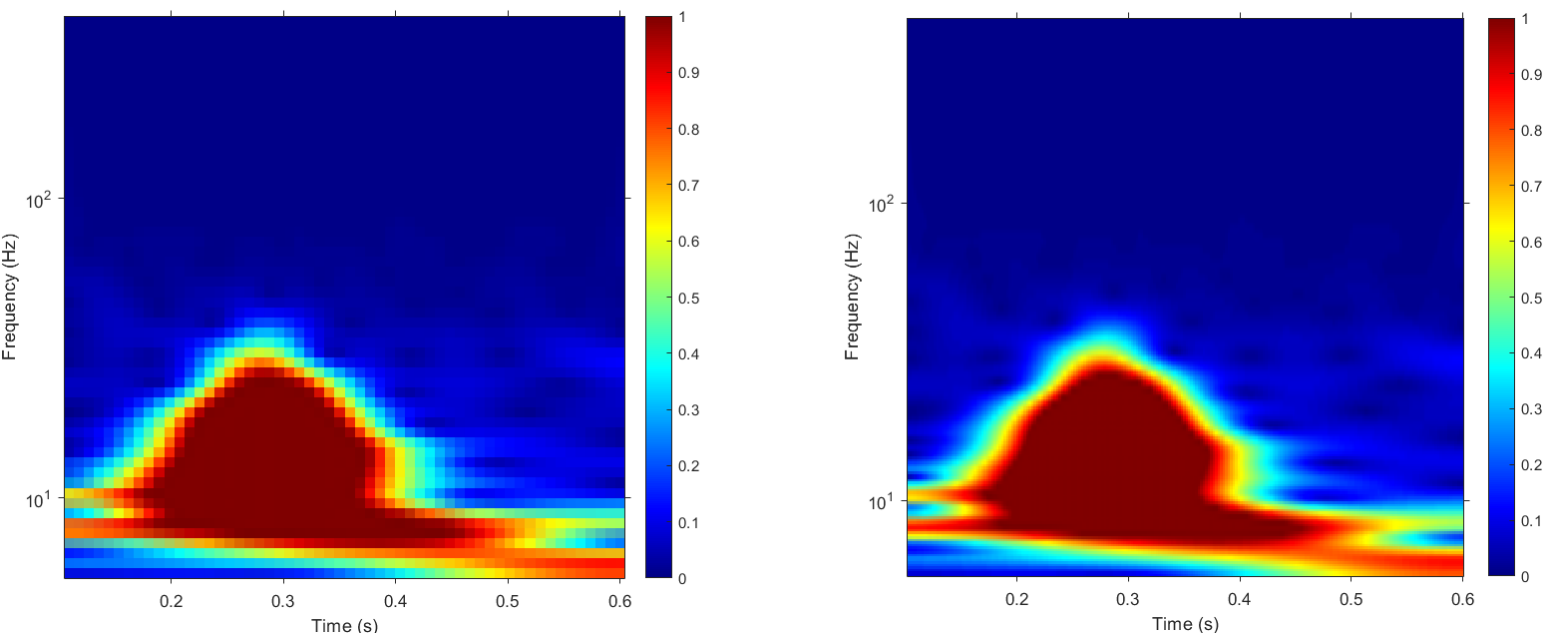
Scalogram representations of a randomly selected electrocardiogram. The scalogram on the left was resized to 56 × 56 pixels, whereas the one on the right was resized to 224 × 224 pixels. The color map is composed of cold and hot colors, varying from blue at the weakest intensity to red at the strongest intensity.

1. **Template Based on Cardiac Cycles**
  The template based on cardiac cycles is obtained through a three-step process: segmentation, normalization, and segment elimination:
  - **Segmentation:** Neurokit2 [50] was used for signal segmentation, resulting in individual heartbeats with 600 samples, 200 before and 400 after the R peaks, in order to mitigate the heart rate variability between subjects. With this algorithm, if the R wave is not present in a cycle, the segmentation of such a cycle is not completed, and so any cycle without an R wave will not be considered for further processing.
  - **Normalization:** The ECG varies over time due to several factors, such as differences in acquisition equipment or the interaction of the subject with it, which may cause differences in signal amplitude. In order to ensure high performance regardless of this, in the present work, each segment is scaled to vary between 0 and 1, according to the min–max normalization method proposed by Irvine et al. [52].
  - **Segment Elimination:** An outlier removal procedure was applied to eliminate the segments that contain substantial amounts of noise and motion artifacts. The algorithm computes the Euclidean distance between all the heartbeat waveforms, and then, it finds, for each subject, the 20 and the 60 cardiac cycles more similar to each other, corresponding to Set 1 and Set 2, respectively. These two sets of segments correspond to the cardiac cycles-based templates. Figure 3 shows the normalized Set 2 of the training cardiac cycles templates of a randomly selected subject.
2. **Template Based on Scalograms**
  The other type of template used was based on scalograms. A scalogram is the absolute value of the convolutional wavelet transform (CWT) coefficients of a signal. In the present paper, CWT based on Morse wavelet with γ=3 and $P^{2}=60$ was used to transform the cardiac cycles to a series of corresponding 2D time-frequency scalogram representations. The scalograms were resized to squared-scalograms of size 56 × 56 and 224 × 224 pixels-called: from now on, Size 56 and Size 224. As for the cardiac cycles, two sets of templates based on scalograms were generated for each subject- Set 1 and Set 2. Figure 4 illustrates the time-frequency scalogram representation of a cardiac cycle from a randomly selected subject.

#### 3.2.3. Dimensionality Reduction

Prior to performing ICA, the total number of templates of each subject were concatenated. In the concatenated configuration, the training and testing sets are represented as a matrix in which the rows represent subject templates and the columns represent features. The training and testing sets have dimension 63 × 188,160 (63 subjects by 56 × 56 × 3 RGB channels × 20 scalograms) and 63 × 3,010,560 (63 subjects by 224 × 224 × 3 RGB channels × 20 scalograms) for scalograms of sizes 56 and 224, respectively, and dimension 63 × 12,000 (63 subjects by 600 × 20 cycles) for cardiac cycles.

Thus, since the dimension of each template is considerably high, performing template matching would be computationally expensive, regardless of the approach used. In order to reduce the dimension of the templates, ICA was applied, using the FastICA algorithm implemented in MATLAB [53], resulting in a matrix with the independent components (ICs) dimensionality $63^{2}$. Thereafter, the enrollment and presentation templates were transformed according to this IC matrix, which significantly minimizes the computational cost.

### 3.3. Identification Algorithm

The identification system was tested using cardiac cycles and scalograms as inputs, with different methodologies. The templates from the first acquisition session comprise the training set, whereas the templates from the second acquisition comprise the testing set.

#### 3.3.1. Identification Based on Cardiac Cycles

For the identification based on cardiac cycles, different classifiers, namely LDA, kNN, DT, and SVM, were implemented in two different configurations: Configuration 20/20 and Configuration 60/60. In the former, classifiers were trained with Set 1 from the training templates (i.e., the templates obtained from the signals of the first acquisition session) and tested with Set 1 from the testing templates (i.e., the templates obtained from the signals of the second acquisition session). In the Configuration 60/60, Set 2 of the training and testing templates were used to train and test the classifiers, respectively. In this section, templates concatenation and dimensionality reduction (DR) were not applied. All the models were fed with normalized and not normalized templates. The classification models were compared based on the following evaluation metrics: accuracy, weighted precision and recall, and F1 score.

#### 3.3.2. Identification Based on Scalograms

The scalograms in Size 56 and Size 224 were given as input to a 15-layer convolution neural network (CNN) and to a Manhattan distance-based 1-NN classifier. The CNN was performed in Configuration 20/20 and Configuration 60/60. For the distance-based algorithm, the inputs were concatenated and scalograms were dimensionally-reduced in Configuration 1/1 and Configuration 1/3. For Configuration 1/1, a subject is correctly identified if their testing template is the most similar to their training template. For Configuration 1/3, a subject is correctly identified if at least two of their testing templates are the most similar to their training template.

### 3.4. Authentication Algorithm

The authentication was performed with a distance-based template matching algorithm. Then, a leave-one-out cross-validation method was performed for impostors testing. The performance of the system was assessed according to two evaluation metrics: accuracy, corresponding to the quotient between the number of subjects correctly authenticated and the number of subjects present in the database, and impostor score, corresponding to the mean of the number of impostors authenticated per subject.

#### 3.4.1. Distance-Based Algorithm

The algorithm computes the differences between the training and testing templates, with Manhattan distance. For Configuration 1/1, a subject is authenticated if the distance between their training and their testing template does not exceed a threshold defined for that subject. For Configuration 1/3, the subject is authenticated if the distances between their training template and, at least, two of the three testing templates are lower than the threshold for that subject.

The threshold was defined individually for each subject, using the distance between the testing template(s) of the subject and the training templates of all the subjects. The threshold for each subject is calculated according to the following equation:

(1) $$T_{i}=\mu_{i}-\sigma_{i},$$

where $\mu_{i}$ and $\sigma_{i}$ are the mean and the standard deviation of the distances per subject, respectively.

#### 3.4.2. Leave-One-Out Cross-Validation

As proposed in the literature [3,33,47], the number of impostors was computed using a leave-one-out cross-validation strategy, which uses each subject as a “test” set and the remaining as the training set.

The algorithm uses a training set with n-1 subjects, with n being the number of subjects present in the database. Then, the Manhattan distances between the template of the “test” subject and the templates of the “train” subjects are computed. These distances are used to calculate the threshold for that subject according to Equation (1). The process is repeated *n* times, changing the testing subject in each iteration. In Configuration 1/1, a subject is authenticated as an impostor of the “test” subject if the distance between their training and testing templates is below the threshold. In Configuration 1/3, a subject is considered an impostor of the “test” subject if the distance between their training and, at least, two of the testing templates is below the threshold.

## 4. Results

### 4.1. Identification Results

#### 4.1.1. Identification Based on Cardiac Cycles

The classifiers were evaluated according to several metrics—accuracy, precision, recall, and F1 score. Table 1 and Table 2 present the results of the evaluation metrics for each of the classifiers, for not normalized and normalized cardiac cycles, respectively.

Whether the cardiac cycles were normalized or not, the four classifiers performed better in Configuration 60/60, since the accuracy and the metrics were higher when using 60 templates to predict the identification of the participants.

For Configuration 60/60, with and without normalization, the classifier that performed best was the LDA, followed by kNN, SVM, and, finally, DT. The LDA yielded an accuracy of 77.87% and 79.37% for not normalized and normalized cardiac cycles, respectively, whereas DT achieved 52.38% and 58.73% without and with normalization, respectively. For Configuration 20/20, the achieved accuracy was lower, but LDA was also the more accurate classifier, showing an accuracy of 74.60% and 69.84% without and with normalization, respectively, whereas DT was the less accurate, with an accuracy of 52.38% for not normalized cardiac cycles and 34.92% with normalization.

The best configuration is the one that uses more templates per subject (Configuration 60/60). Our results suggest that by normalizing templates, the accuracy increases for all classifiers, except for SVM, where the accuracy slightly decreases from 61.90% to 58.73% when normalization is performed.

#### 4.1.2. Identification Based on Scalograms

The scalogram-based templates were tested as inputs for an identification algorithm following two methodologies: a neural network and a distance-based algorithm.

1. Identification Based on Neural Networks
  Table 3 presents the performance of the proposed 15-layer CNN based on the obtained accuracies.
  According to the results in Table 3, the CNN classifier is more accurate at identifying subjects if the number of inputs per subject is greater. These results are in agreement with the results obtained for the cardiac cycles, since Configuration 60/60 leads to the highest accuracies. The accuracies achieved with and without normalization are very similar, differing only 1.59% (i.e., 1 subject) in Configuration 20/20 of Size 56 and in both configurations of Size 224. Regarding the size of the scalograms, the optimal configuration for Size 56 presented an accuracy of 68.25% at identifying subjects, whereas for Size 224, an accuracy of 69.84% was obtained.
2. Identification based on Distance Metrics
  The scalograms were tested with a Manhattan distance-based 1-NN in a concatenated configuration and with reduced dimensionality. Table 4 presents the results obtained with the considered distance-based algorithm.
  The results in Table 4 show that for Size 56, not normalized scalograms lead to higher accuracies when compared to normalized scalograms for both configurations. Moreover, for not normalized scalograms, Configuration 1/3 allows the algorithm to identify subjects more accurately. For Size 224, the opposite happens. Normalized scalograms and Configuration 1/1 optimize the performance of the system.

### 4.2. Authentication Results

**Figure 5:**
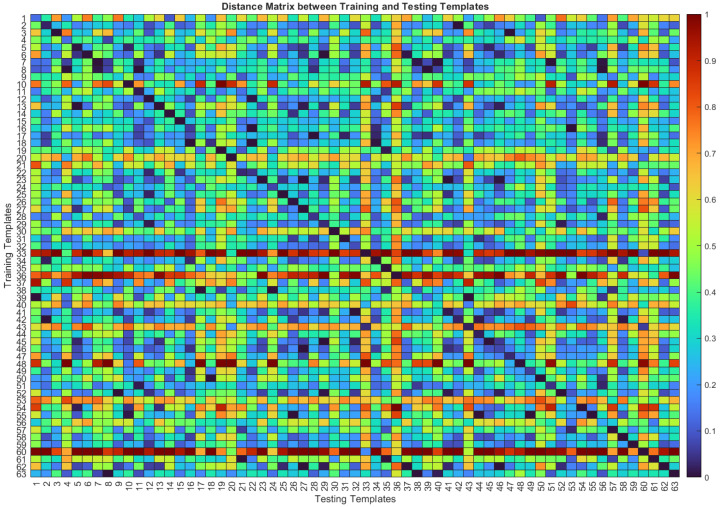
Distance matrix between training and testing templates based on not normalized cardiac cycles with DR in Configuration 1/1. The cold colors (blue) correspond to smaller distances, whereas warm colors (red) correspond to larger distances.

The distance-based template-matching algorithm computes the Manhattan distance between the training and testing templates for each subject. Figure 5 shows the distance matrix between training and testing templates for one randomly selected configuration. The figure shows a diagonal line, in which the colors from all the entries are blue. This means that the distances between the testing and training templates from each subject are, in general, small. This pattern is an indicator that this algorithm would be a promising approach to authenticate subjects, since low distances are expected to be below the threshold set for each subject, which is what happens in the case of authentication.

**Figure 6:**
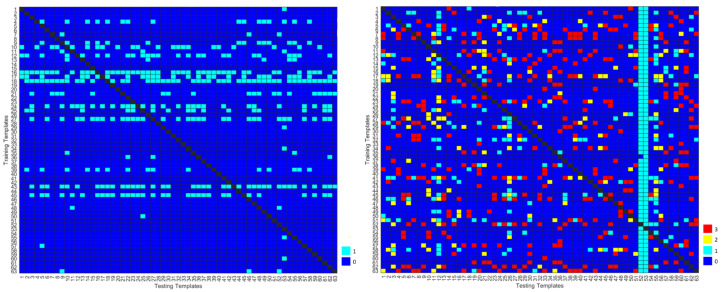
Number of authenticated impostors when using not normalized cardiac cycles with DR, in (Configuration 1/1 and Configuration 1/3, on the left and right, respectively). The colors represent the number of templates of each subject that are authenticated as impostors, namely 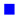 and 0 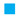 and 1 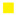 and 2 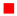 and 3.

The distance-based template matching algorithm was also evaluated by assessing the capacity of the system to reject potential impostors. Figure 6 shows the authenticated impostors for each subject for the not normalized cardiac cycles with DR. The diagonal lines are black because each entry corresponds to a single subject’s training and testing templates, and a subject cannot be an impostor of themselves. The impostors of each subject are represented in light blue in the vertical line corresponding to each subject.

#### 4.2.1. Authentication Based on Cardiac Cycles

Table 5 and Table 6 present the accuracies and impostor scores, respectively, obtained when using cardiac cycles with and without DR. Whether normalization and DR are performed or not, results are very similar for Configuration 1/1 and Configuration 1/3. The accuracies obtained were higher for not normalized cardiac cycles (90.48% and 88.80% without and with DR, respectively), whereas the lowest impostor scores were obtained for normalized cardiac cycles (7.56% without DR).

#### 4.2.2. Authentication Based on Scalograms

Opposite to what happened for the cardiac cycles, scalograms were only used with reduced dimensionality by applying ICA, due to their significantly large size. The number of ICs that optimized the algorithm was 63. Table 7 shows the obtained accuracies, whereas Table 8 shows the corresponding impostor scores when the DR was performed with 63 ICs.

In Table 7, regarding not normalized scalograms of both sizes, the results showed almost no differences in the accuracy and impostor score when comparing Configuration 1/1 to Configuration 1/3. However, the accuracy was higher for Size 224, and the impostor score was lower. When using the normalized scalograms of Size 56, Configuration 1/3 reached a higher accuracy, 98.42%. Size 224 did not cause differences among configurations, reaching an accuracy of 93.65% and an impostor score of approximately 14.55%.

In Table 8, for Size 56, the impostor scores achieved using Configuration 1/1 and Configuration 1/3 were higher for not normalized than for normalized scalograms (16.21% against 14.34%). For Size 224, the impostor scores were slightly higher for both configurations using not normalized scalograms. When combining the two evaluation metrics, the best performance of the distance-based authentication system is achieved for normalized scalograms resized to 56 × 56 in Configuration 1/3, with an accuracy of 98.42% and an impostor score of 14.34%.

## 5. Discussion

Concerning the identification based on cardiac cycles, the results suggest a better performance of the identification system in Configuration 60/60. Thus, more templates capture the variability of the subject’s heartbeat better, which is mainly due to the incorporation of noisier cardiac cycles in the training process. If the classifier is only trained with the most similar cardiac cycles, which happens in Configuration 20/20, it will probably fail more often when classifying noisier testing cycles.

Despite the configuration, LDA and DT showed the best and the worst performance, respectively. To the best of our knowledge, most studies on ECG biometrics use LDA for dimensionality reduction rather than for classification [19,31,33], being [27] an exception. In the current state-of-the-art, very few studies investigated the application of a DT for biometric identification purposes. The low accuracies obtained may be due to the fact that DT are prone to overfitting, meaning that they can be overcomplex and, consequently, not able to generalize well from training data, especially if the testing and training data are very different, which is likely to happen when data are acquired in two different acquisitions separated by 3 months.

Our results suggest that by normalizing templates, the accuracy increases for all classifiers, except for SVM. Therefore, normalization proved to be an essential step to distinguish subjects and consequently identify them correctly. To the best of our knowledge, most studies, due to the inherent heartbeat waveform variability, performed normalization in order to obtain amplitude and time-invariant characteristics applicable to biometric purposes [54].

Thereby, for the optimal configuration, which corresponds to the use of normalized cardiac cycles in Configuration 60/60 with LDA, 50 subjects (79.37%) were correctly identified by the system. As mentioned above, Shen et al. [27] also used LDA as a distance classifier, achieving an accuracy of 96% for 100 subjects and 95.3% for 168 subjects. Our algorithm underperformed in this study; the reason for this was that the ECG data in [27] were collected in an on-the-person approach with 12 leads, whereas our data were from an off-the-person acquisition set-up, making signals more susceptible to noise and interference and consequently more prone to misclassification.

For the identification based on scalograms, the results suggest that neural networks do not need normalization to perform an accurate identification of subjects, since the accuracies obtained with and without normalization are very similar for the scalograms of both sizes. Configuration 60/60 reached higher accuracies, meaning that these results are in agreement with those obtained for the cardiac cycles. The size of the scalograms did not influence the accuracy of the system for most configurations; despite the number of pixels of the scalogram, the classifier can distinguish subjects. Moreover, since size 224 × 224 is four times greater than size 56 × 56, the computational time of the former is also four times greater. Thereby, considering the accuracy and computational time of the system, using scalograms Size 56 represents the best trade-off.

For the distance-based algorithm, scalograms of Size 56 reached a higher accuracy when not normalized, whereas scalograms of Size 224 performed better when normalized. When the number of pixels is reduced, some information is lost. When performing normalization, information on the ECG voltage is also lost, making the scalograms more similar to each other. The results proved that if some information is lost by reducing the number of pixels of the scalograms (Size 56), the system needs the templates to be as different as possible from each other in order to accurately distinguish them. Hence, it was verified that normalization is advantageous when more pixels are considered. Since the system has difficulties at matching scalograms of Size 224, it was expected that the system would struggle when trying to classify noisier segments, which was observed in Configuration 1/3. For Size 56, we observed the opposite; since there are fewer pixels, noisier templates will help to distinguish subjects.

The optimal configuration for the distance-based algorithm, which used not normalized scalograms of Size 56 in Configuration 1/3, reached an accuracy of 58.73%. Since for the neural network, the optimal configuration achieved an accuracy of 68.25%, corresponding to the correct identification of 43 subjects, the neural network is the most accurate method to identify subjects with templates based on scalograms. According to our literature review, Byeon et al. [55] also proposed an intelligent deep model based on scalograms of electrocardiogram signals for biometrics, reaching an accuracy of 87.5%. Our method underperformed theirs; however, our data were collected in an off-the-person approach, and we used two separate acquisition sessions (that took place on different days) for either training or testing of the classifiers, which is a more similar scenario to a real application.

Regarding the authentication based on cardiac cycles, results showed that both accuracies and impostor scores are very similar for both configurations, meaning that the number of cardiac cycles used to authenticate a subject has little influence on the performance of the system. The lowest impostor score was obtained with normalized cardiac cycles, and the highest accuracy was obtained without normalization. Thus, normalization proved to be an essential procedure to efficiently reject potential impostors, but it limits the capacity of the system to correctly authenticate the subjects.

When comparing the two evaluation metrics, we can conclude that not normalizing the cardiac cycles is a better option for the authentication system. However, a conclusion cannot be drawn on whether DR was advantageous or not based on the accuracy and on the resulting impostor score. For the optimal configuration, the system was able to authenticate 57 subjects (90.48%) and reject 54 potential impostors (approximately 13%). According to our literature review, Arteaga-Falconi et al. [10] used a similar database (off-the-person acquisitions and two acquisition sessions in different days) for the authentication of individuals. Despite being based on distances, their authentication algorithm is quite different as they proposed a hierarchical validation scheme that evaluates each feature individually. They reached an accuracy of 81.82% using fiducial features based on cardiac cycles. Thus, our algorithm outperformed this study.

For the authentication based on scalograms, results were similar for both sizes of scalograms, both configurations and whether normalization was performed or not. Thus, we can conclude that the scalograms-based template is suitable to train this distance-based algorithm, since for the best configuration, 62 subjects were authenticated (98.42%) and 53 potential impostors were rejected (14.34%). Even though the computational time of each method was not calculated, both the scalogram generation and the dimensionality reduction are time-consuming processes. To the best of our knowledge, no studies used scalograms to perform authentication; instead, we only found studies pursuing identification. Scalograms proved to be quite a promising approach for the authentication task.

## 6. Conclusions

Research on ECG signals has advanced significantly since its clinical roots to novel application domains in areas as diverse as biometric recognition. Our research has evaluated the feasibility of ECG as a biometric for individual identification and authentication tasks.

In our study, we thoroughly compare and evaluate several approaches and methodologies in different phases of the biometric system, and we find the optimal solutions according to the results obtained. All the methodologies were tested in an across-session modality, as biometric systems must function for an indefinite amount of time.

Concerning the template generation procedure, two types of templates were considered: cardiac cycles and scalograms of two different sizes: Size 56 and Size 224. Results from the various template-matching methods showed a better performance for Size 56, meaning that templates sized 224 × 224 may contain too much detailed information from each subject that it is difficult to find a signal that matches so many characteristics. Moreover, the generation of scalograms is a computationally time-consuming process and the smaller the size of the scalograms, the less time it takes to compute.

We also analyzed the influence of the number of templates, per subject, on the accuracy of the biometric system. The results showed that this is not a linear issue, as for some configurations, the performance is better when noisier templates are included (more templates per subject), while for other configurations, it is better to use fewer templates.

For the identification with cardiac cycles, the optimal performance was achieved with LDA, whereas for the identification with scalograms, the optimal performance was achieved with the neural network. The best identification system was based on cardiac cycles, both in terms of accuracy and computational time. For the authentication system, the use of cardiac cycles allowed a better rejection of impostors, whereas the scalograms allowed a more accurate authentication of subjects. Thus, future work should investigate the computational time of each process to conclude about the most accurate method, taking into account all the important characteristics of a biometric system.

Further experiments should also be completed to build a system based on a larger database, comprising users of all ages, abnormal ECG data, and the long span of time interval between ECG recordings in order to simulate a more realistic biometric system.

## Figures and Tables

**Table 1:** Comparison of the performance of the identification task based on classifiers for not normalized cardiac cycles.

Not Normalized Cardiac Cycles					
Configuration	Classifier	Accuracy	Weighted Average		
			Precision	Recall	F1-Score
Configuration 20/20	LDA	74.60%	68.34%	67.70%	64.48%
	kNN	60.32%	53.08%	52.30%	49.56%
	DT	52.38%	42.04%	41.90%	38.18%
	SVM	60.32%	52.84%	54.84%	50.67%
Configuration 60/60	LDA	77.78%	71.88%	71.40%	68.23%
	kNN	63.49%	54.52%	51.72%	49.34%
	DT	52.38%	42.40%	40.21%	37.68%
	SVM	61.90%	57.81%	52.96%	50.08%

**Table 2:** Comparison of the performance of the identification task based on classifiers for normalized cardiac cycles.

Normalized Cardiac Cycles					
Configuration	Classifier	Accuracy	Weighted Average		
			Precision	Recall	F1-Score
Configuration 20/20	LDA	69.84%	64.19%	62.86%	58.49%
	kNN	57.14%	51.37%	50.63%	47.09%
	DT	34.92%	32.52%	29.68%	28.89%
	SVM	52.38%	49.70%	46.27%	44.27%
Configuration 60/60	LDA	79.37%	67.99%	69.13%	65.21%
	kNN	68.25%	52.55%	53.07%	49.54%
	DT	58.73%	40.12%	38.60%	36.27%
	SVM	58.73%	50.52%	49.76%	46.05%

**Table 3:** Comparison of the performance of the identification algorithm based on 15-layers CNN between not normalized and normalized scalograms of Size 56 and Size 224.

	Not Normalized Scalograms	Normalized Scalograms	
Size of Scalograms	Accuracy		Configuration
Size 56	65.08%	63.49%	Configuration 20/20
	68.25%	68.25%	Configuration 60/60
Size 224	61.90%	63.49%	Configuration 20/20
	69.84%	68.25%	Configuration 60/60

**Table 4:** Comparison of the performance of the identification algorithm based on distance metrics between not normalized and normalized scalograms Size 56 and Size 224.

	Not Normalized Scalograms	Normalized Scalograms	
Size of Scalograms	Accuracy		Configuration
Size 56	50.79%	47.62%	Configuration 1/1
	58.73%	52.38%	Configuration 1/3
Size 224	47.62%	53.97%	Configuration 1/1
	44.44%	46.03%	Configuration 1/3

**Table 5:** Comparison of the accuracy of the authentication algorithm between not normalized and normalized segments, with and without DR.

	Not Normalized Cardiac Cycles		Normalized Cardiac Cycles	
Configuration	Accuracy			
	Without DR	With DR	Without DR	With DR
Configuration 1/1	90.48%	88.89%	57.14%	79.37%
Configuration 1/3	90.48%	87.30%	55.56%	77.78%

**Table 6:** Comparison of the impostor score of the authentication algorithm between not normalized and normalized segments, with and without DR.

	Not Normalized Cardiac Cycles		Normalized Cardiac Cycles	
Configuration	Impostor Score			
	Without DR	With DR	Without DR	With DR
Configuration 1/1	13.21%	12.93%	7.71%	13.80%
Configuration 1/3	13.06%	12.95%	7.56%	13.57%

**Table 7:** Comparison of the accuracy of the authentication algorithm between not normalized and normalized scalograms Size 56 and Size 224.

	Not Normalized Scalograms	Normalized Scalograms	
Size of Scalograms	Accuracy		Configuration
Size 56	92.06%	92.06%	Configuration 1/1
	92.06%	98.42%	Configuration 1/3
Size 224	93.65%	93.65%	Configuration 1/1
	93.65%	93.65%	Configuration 1/3

**Table 8:** Comparison of the impostor score of the authentication algorithm between not normalized and normalized scalograms Size 56 and Size 224.

	Not Normalized Scalograms	Normalized Scalograms	
Size of Scalograms	Impostor Score		Configuration
Size 56	16.21%	14.34%	Configuration 1/1
	16.21%	14.34%	Configuration 1/3
Size 224	15.16%	14.59%	Configuration 1/1
	14.97%	14.52%	Configuration 1/3

## Data Availability

Not applicable.
